# Towards full-stack deep learning-empowered data processing pipeline for synchrotron tomography experiments

**DOI:** 10.1016/j.xinn.2023.100539

**Published:** 2023-11-16

**Authors:** Zhen Zhang, Chun Li, Wenhui Wang, Zheng Dong, Gongfa Liu, Yuhui Dong, Yi Zhang

**Affiliations:** 1 National Synchrotron Radiation Laboratory, University of Science and Technology of China, Hefei 230029, China; 2 Beijing Synchrotron Radiation Facility, Institute of High Energy Physics, Chinese Academy of Sciences, Beijing 100049, China

## Abstract

Synchrotron tomography experiments are transitioning into multifunctional, cross-scale, and dynamic characterizations, enabled by new-generation synchrotron light sources and fast developments in beamline instrumentation. However, with the spatial and temporal resolving power entering a new era, this transition generates vast amounts of data, which imposes a significant burden on the data processing end. Today, as a highly accurate and efficient data processing method, deep learning shows great potential to address the big data challenge being encountered at future synchrotron beamlines. In this review, we discuss recent advances employing deep learning at different stages of the synchrotron tomography data processing pipeline. We also highlight how applications in other data-intensive fields, such as medical imaging and electron tomography, can be migrated to synchrotron tomography. Finally, we provide our thoughts on possible challenges and opportunities as well as the outlook, envisioning selected deep learning methods, curated big models, and customized learning strategies, all through an intelligent scheduling solution.

## Public summary

• Tomography experiments at future synchrotron beamlines face exascale big data challenges.
• Deep learning is employed for various data processing tasks at current synchrotron tomography beamlines.
• Future comprehensive and elongated data analysis processes require a full-stack deep learning pipeline.
• The data-driven full-stack deep learning pipeline based on an intelligent scheduling center (ISC) holds the key to solve the big data challenges.

## Graphical abstract

**Figure:**
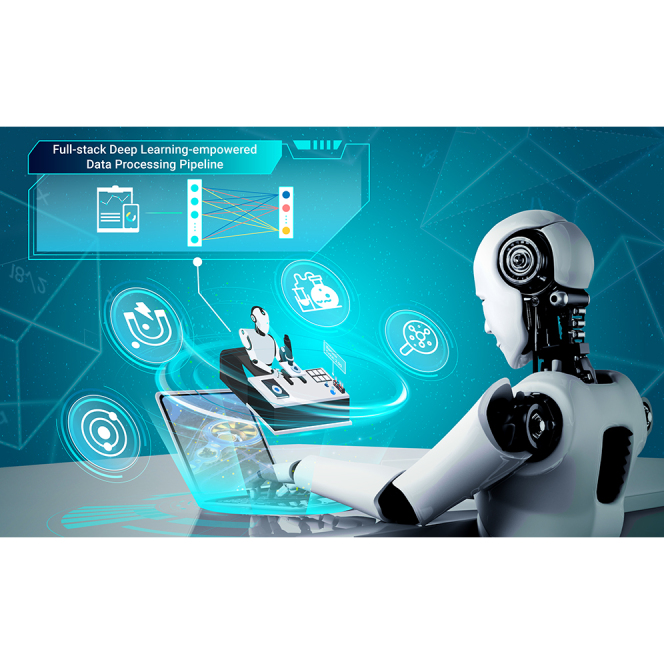

## Introduction

The methodology of tomography is a three-dimensional (3D) characterization technique capable of revealing internal structures and functions of matter using various probe types, such as X-rays, electrons, neutrons, etc. Tomography has been widely adopted in various scientific fields since its emergence in the early 20th century. In biology,^1^^,^^2^^,^^3^^,^^4^^,^^5^^,^^6^^,^^7^ tomography unveils 3D assembly and interaction mechanisms from macromolecules, organelles and cells to tissues and living organisms, making it a powerful tool for uncovering the mysteries of life. In medical science,^8^^,^^9^^,^^10^^,^^11^^,^^12^ tomography facilitates internal examination of lesion areas, aiding in the subsequent diagnosis and treatment process. In materials science,^13^^,^^14^^,^^15^^,^^16^^,^^17^^,^^18^ tomography, when combined with *in situ* methods, is used to analyze structures, identify defects, and uncover the dynamics within materials, greatly benefitting the discovery and study of new-generation functional materials.

The boundary of tomography is pushed further with the commissioning of large scientific research facilities, such as new-generation synchrotron light sources,^19^^,^^20^^,^^21^ which greatly improves the ability to characterize matter at extreme spatial and temporal resolution. This improvement is mostly due to the ultra-bright, time-resolved, and highly coherent nature of X-ray sources generated in the beamlines of new-generation synchrotron facilities, which enables synchrotron tomography. Advanced imaging techniques, such as ptychography^22^ and holography,^23^ are being developed to further promote the spatial resolution to below10 nm. At the ID16A^24^ beamline in European Synchrotron Radiation Facility (ESRF), the spatial resolving capability of X-ray nano-holotomography experiments is reaching cryoelectron microscopy (cryo-EM) levels^24^ with an incomparable time resolving advantage for *in situ* characterization of large millimeter-size samples. To image even larger samples, the hierarchical phase-contrast tomography (HiP-CT)^25^ technique offered by the BM05 beamline at ESRF has demonstrated the capability to image the entire lung of a coronavirus disease 2019 (COVID-19) patient with 1-μm resolution, which unlocks the resolving power on subcellular features. In modern synchrotron tomography, there is a rising interest in multimodal characterizations, which facilitates a better understanding of structural and functional relationships. For example, at nanoprobe beamlines such as Hard X-ray Nanoprobe (HXN; Brookhaven National Laboratory)^26^ and P06 (Deutsches Elektronen-Synchrotron),^27^ nanoscale X-ray spectroscopy (fluorescence, absorption near-edge structure), diffraction, ptychography, and tomography experiments can be performed simultaneously to acquire both chemical and structural information; small-angle X-ray scattering tensor tomography and wide-angle X-ray diffraction tensor tomography are often combined with high-resolution absorption tomography experiments to examine the orientation and strain information in textured samples. Another important trend is dynamic tomography, where *in situ* or dynamic experiments are being undertaken as routine in many beamlines. For example, the TomoCAT beamline (Paul Scherrer Institute)^28^ is capable of providing serial tomography characterization techniques at a rate of 20 Hz.

These changes in synchrotron tomography inevitably lead to explosive growth in data volume and dimension as well as elongated processing pipelines, imposing a tremendous burden on the data processing end.^29^ Some beamlines are expected to generate petabytes (PBs) of data per day, becoming some of the largest data sources for scientific experiments. The demand on real-time analysis will be more imperative than ever. Therefore, it is essential to address the big data challenge, optimize the tomographic data processing pipeline, and ultimately improve experimental characterization capabilities because conventional techniques are still struggling to cope with such workloads.

Today, scientific research is entering the “fourth paradigm,”^30^ powered by numerous data-driven approaches. Among them, deep learning (DL) techniques have been widely acknowledged as a highly accurate and efficient data processing method compared with conventional techniques. Over the last few decades, DL-based network models have achieved outstanding performance on various downstream tasks, such as classification, object recognition, instance segmentation, image generation, and more. From the emergence of artificial neural networks (ANN) based on multilayer perceptron (MLP) architecture in the 1990s to deep neural networks constructed by convolutional neural network (CNN) and today’s transformer architecture that showcases superior feature extraction capabilities, DL architectures with higher efficiency and accuracy have proliferated. One of the advantages of DL is the ability to use massive amounts of data for learning, which maps the principles between inputs and outputs while overcoming problems caused by formulaic solutions. Therefore, researchers are exploring the possibilities of applying the “scientific big data + DL” paradigm to synchrotron tomography. Integrating DL into the synchrotron tomography data processing pipeline is believed to be the ultimate solution to tackle the challenges encountered in high-resolution, multimodal, cross-scale, and dynamic experiments.

**Figure 1:**
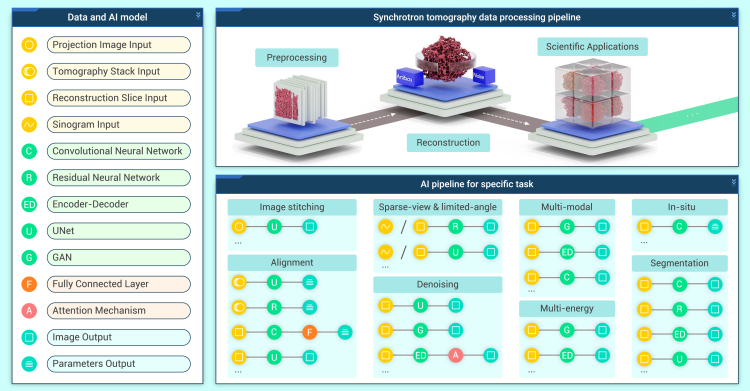
Overview of DL in the STDPP DL is tightly integrated with each part of the STDPP to improve experimental efficiency and accuracy.

Taking 3D reconstruction as a pivotal module, DL applications on the synchrotron tomography data processing pipeline can be categorized as three workloads in succession (Figure 1): image processing before reconstruction (preprocessing), 3D reconstruction optimization, and scientific application-oriented data processing on reconstructed data. The preprocessing workload usually involves image stitching and a series of correction tasks to tackle the misalignment of the projection images due to system vibrations and offsets. The optimization process usually concentrates on defect mitigation, specifically on optimization of the 3D reconstruction process for sparse-view datasets, as well as denoising on reconstructed volumes. Finally, scientific application-oriented data processing on reconstructed data task directly relates to specific scientific goals.

This review aims to provide a comprehensive overview of DL applications in the synchrotron tomography data processing pipeline (STDPP). It will present and discuss each element of the pipeline, providing guidance on tomographic data processing for both inexperienced and skilled researchers in the data-driven era. In addition, other data-intensive research fields, such as medical imaging and electron tomography (ET), are similar to synchrotron tomography in terms of application scenarios. With DL being more thoroughly studied in these two fields, well-established methods will be included in this review. We will first provide an overview of the evolution of DL techniques. Then, we concentrate on DL applications in the preprocessing stage of the STDPP, summarizing DL achievements and drawbacks in this phase. Next, we discuss how DL, combining with techniques used in other scientific fields, can help handle the reconstruction optimization problem. Additionally, we deal with DL applications in the scientific application-oriented data processing tasks after 3D reconstruction, with an emphasis on region of interest (ROI) segmentation tasks, multimodal tomography, multienergy tomography, and *in situ* tomography. Finally, we discuss prospective challenges, opportunities, and outlooks DL is encountering in the STDPP by proposing three critical components: synchrotron tomography big model, intelligent scheduling center, and single-facility learning and long-term learning.

## Overview of the evolution of DL techniques

**Figure 2:**
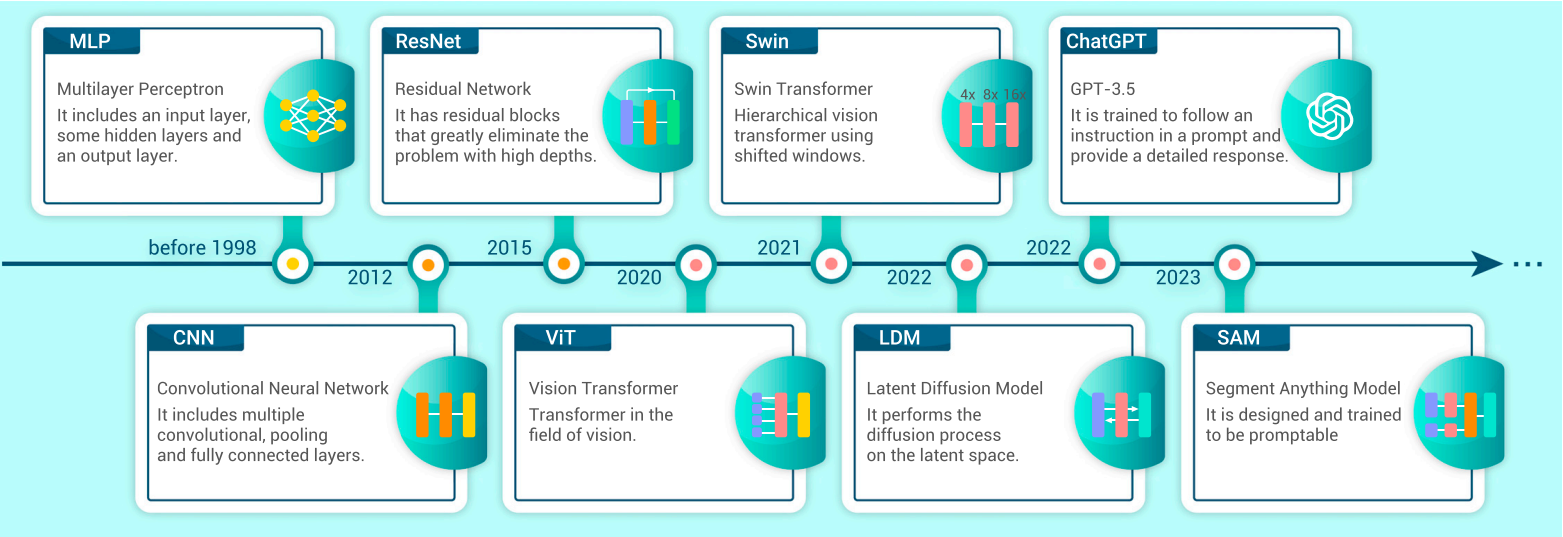
Overview of the development of DL The development of new DL technologies has driven advances in the field of synchrotron tomography.

DL has been a research hotspot in machine learning since its emergence in the 1980s. Early DL models were often regarded as ANNs with an MLP architecture (Figure 2). However, due to the massive number of learning parameters and the vanishing gradient problems, the stability of ANNs during the training phase could not always be guaranteed, and their performance in image processing tasks often fell short. In 1998, CNNs were first introduced in machine vision applications with the introduction of LeNet.^31^ CNNs typically consist of multiple layers, such as convolutional, pooling, and fully connected (FC) layers, which effectively alleviate the problem of parameter redundancy and training difficulties. In 2012, Krizhevsky et al.^32^ proposed AlexNet, which has deeper structures and uses rectified linear units (ReLU)^33^ as an activation function instead of Sigmoid. The number and quality of extracted features using AlexNet significantly surpassed those using manual approaches, making AlexNet the inception of modern CNNs.

In the following years, numerous meticulously designed CNN architectures emerged^34^^,^^35^^,^^36^ with deeper network structures, but training difficulties and the vanishing gradient problem still persist, and the performance is nearly reaching the limit. However, in 2015, He et al.^37^ proposed ResNet, which dramatically improved the performance of CNNs. ResNet uses residual blocks by summating input and output features to fuse information, circumventing vanishing gradients incurred by overly deep network architectures. Today, residual blocks are the primary DL network modules applied in synchrotron tomography.

Recently, the performance of DL has excelled in natural language processing (NLP) tasks using transformer architectures. In 2020, a research team from Google proposed Vision Transformer (ViT)^38^ and applied transformer architectures to computer vision applications. Subsequently, the Swin-transformer^39^ network has pushed the performance limit of CNNs even further. Plus, the Latent Diffusion Model (LDM),^40^ proposed in 2022, achieved a performance breakthrough in image generation tasks using a generative adversarial network (GAN).^41^ Through progressive development, the number of parameters in DL networks has dramatically increased, and computer vision is finally entering the big-model era. Even today, newer models are actively proposed, such as ChatGPT and Segment Anything Model (SAM),^42^ and the performance of DL models based on transformers still has not reached its limit,^38^ with rapid development still ongoing.

From the basic MLP, the prevailing CNN, and highly effective ResNet to the more recent ViT, LDM, and ChatGPT, DL has and will continue to demonstrate its potential at each stage of the STDPP: image processing before reconstruction, 3D reconstruction optimization, and scientific application-oriented data processing.

## Image processing before reconstruction

**Figure 3:**
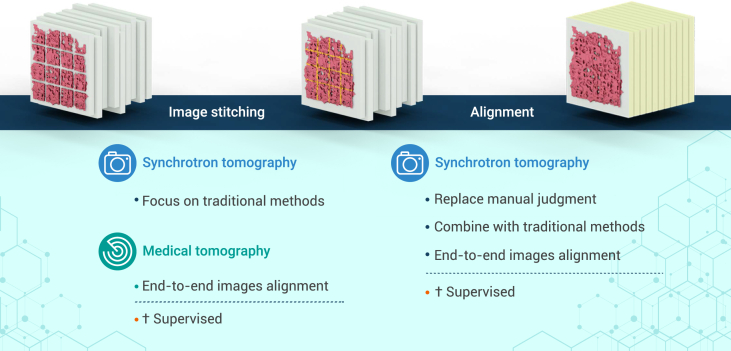
Combination of DL and image processing before reconstruction DL provides excellent pre-processed projection images for 3D reconstruction.

To begin, we will discuss DL’s applications on the preprocessing workloads before 3D reconstruction, which include image stitching within each projection in scanning tomography experiments and image alignment between different projections (Figure 3).

### Image stitching

In experiments like scanning transmission X-ray microscopy (STXM), only a small portion of the whole 2D projection is captured for each single exposure due to the limited field of view (FoV), and the scanned regions need to be stitched together to obtain a full-projection image. Direct stitching within the scanning area can cause discontinuities and distortions due to drift and jitter of X-ray beams as well as motion inaccuracy from the sample itself. The conventional correction strategies that are currently used, such as feature-based^43^ and cross-correlation^44^ methods, limit the accuracy and stability of the stitched images.

In the medical field, similar pixel drifts and image distortions are also present in Optical Coherence Tomography (OCT) retinal vascular images, which suffer from internal jitters within the imaging system and disturbances caused by the patient. DL methods have been developed to correct for these distortions in OCT images. Qin et al.^45^ proposed a DL distortion correction network in 2021. They utilized a modified UNet architecture to construct a mapping from distorted images to reference images. The modified model demonstrated competence in complicated tasks such as image distortion recovery. Through supervised learning, distorted images could be corrected to near-ground-truth quality, and similar approaches could be explored for synchrotron tomography. However, unlike OCT images, which have publicly available paired datasets such as OCTA-500,^46^ synchrotron radiation computed tomography (SR-CT) lacks sufficient accessible datasets. Moreover, the sample types examined in SR-CT are much more diverse; hence, the training dataset needs to cover a large variety of sample types. Dataset construction is therefore critical in future distortion correction tasks, particularly when real-world data collection is becoming an arduous and time-consuming task within limited beamtime. Because the mechanisms behind distortion are usually uniform and predictable for specific beamlines, one possible solution for data construction is simulation using well-established distortion mechanisms.

### Alignment

Aligning the projection images is another crucial step before reconstruction and aims to correct the misalignments between projection images that can result in artifacts on reconstructed data. Misalignments are generally inevitable in high-resolution tomography experiments due to mechanical instabilities or positioning errors, whether for full-field tomography methods or scanning tomography combined with ptychography, spectroscopy, or diffraction methods. The artifacts are often less obtrusive in microscale synchrotron tomography systems. However, for methodologies performing at nanoscale, the drifting effects would be substantially magnified. When positioning accuracy enters the nanometer regime, the system can no longer be corrected merely by hardware upgrades.

A mechanical system’s instability in synchrotron tomography arises from various aspects, such as motion inaccuracy of the sample and drift and vibration of the imaging system during the long acquisition process. These effects are usually coupled in the postprocessing phase, making the alignment task even more demanding. Conventional methods, like center of mass (CoM),^47^ cross-correlation,^48^^,^^49^ and iterative techniques,^50^^,^^51^^,^^52^^,^^53^^,^^54^ are often applied for tomography stack optimization, but all have their limitations, mostly on accuracy and efficiency. Correcting coupled or mixed types of motion inaccuracy, drift, and vibration in a faster and more accurate fashion is demanding, and extensive research is being undertaken on DL image alignment in the STDPP.

To mitigate instrument instability, in 2016, Yang et al.^55^ proposed a CNN model that calibrates the center of rotation (CoR) in synchrotron tomography. The architecture used was even simpler than that of AlexNet, comprising only two consecutive feature extraction modules. The model distinguishes reconstructions corresponding to correct or incorrect CoRs.^55^ The results matched the performance achieved by human estimation. Most importantly, such a network can be embedded naturally into a data processing pipeline, enabling automatic correction capabilities on rotation axes.

For the more complicated correction task involving both sample drift and detector position inaccuracies, DL can be applied either independently or jointly by combination with conventional techniques, where DL tackles the image alignment problem with an end-to-end correction scheme. In 2020, Topal et al.^56^ proposed a CNN-based DL network to address the component’s thermomechanical instability, where CoR misalignment and detector position inaccuracy happen simultaneously on a ball bearing sample. The network performs segmentation and localization of the same feature-rich regions across different projections. Correction is then made on the CoM using segmented regions. Additionally, in 2022, Fu et al.^57^ proposed a more advanced UNet architecture that segments a common feature area from a series of nano-resolution, full-field transmission X-ray microscopy (nano-CT) stacks acquired from various projection angles. These networks compensate for drift by using the center of a common feature area. Thanks to such advances, estimating sample motion no longer depends on manually added feature points (e.g., using gold particles). Due to the similarity of different instance segmentation problems, advanced instance segmentation networks in computer vision can be naturally migrated into synchrotron tomography. For applications using solely DL methods, in 2021, Fu et al.^58^ proposed a residual network to correct image jitter present in nano-CT. Unlike using original projection images as network input, this model combines the original projections with the reconstructed back-projection images as input. The final output measures the amount of motion compensation of the tomography stack. The reconstructed slices of a battery cathode particle also present better contrast with more detailed internal structures unveiled.

However, due to complex, diverse, and unpredictable sample environments, the generalizability of data processing models in these complicated correction tasks remains a critical concern, mostly owing to the requirement for an immense amount of training data, which can be hardly obtained currently. To improve the model’s generalization capability, in 2022, Liu et al.^59^ designed the multiscale dense U-Net. They adopted data augmentation techniques such as pruning, scaling, shifting, and rotating. They also used MIMO-Unet^60^ architecture, which addresses the task of multiscale fusion by using images with distinctive scales as input to provide abundant prior information. The performance and robustness of the entire network were improved by combination with embedded dense blocks.^61^ When encountering other sample types, the network can still obtain good alignment results with only a small amount of training data provided. However, the problem of data scarcity encountered in supervised learning still exists, and alternate schemes, such as unsupervised or self-supervised learning, will be more suitable to improve models’ generalizability. For instance, the pretext task in contrastive learning^62^^,^^63^^,^^64^^,^^65^^,^^66^ may be used to replace the laborious manual labeling procedure so that the network can be self-trained successfully.

## 3D reconstruction optimization

**Figure 4:**
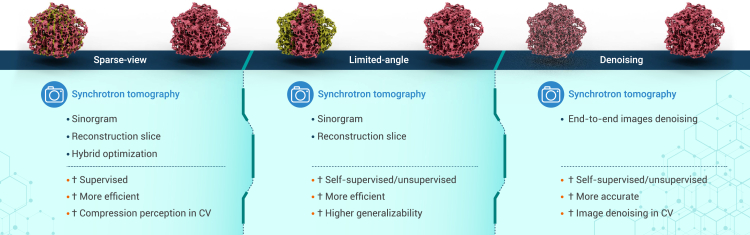
Combination of DL and 3D reconstruction optimization DL is used to improve the quality of reconstructed slices, especially in scenarios where data acquisition is impacted by factors such as sparse view, limited angle, and high noise.

We will now discuss DL applications in the 3D reconstruction stage, focusing on three subjects: optimization of 3D reconstruction for sparse-view acquisition, limited-angle acquisition, and denoising on reconstructed volumes or images (Figure 4).

### Sparse view

To track the 3D structural and functional transformation of the examined object in *operando* or *in situ* experiments, sparse-view acquisition is often employed to speed up the measurement process or alleviate radiation damage. Due to the insufficient images acquired, streak artifacts (Figure 4, green area in sparse view) are prominent on reconstructed tomography slices. Traditional methods usually include compressed sensing approaches that progressively recover data from sparsely acquired images, which is an iterative and time-consuming process. Hence, DL approaches are being applied to optimize sparse-view synchrotron tomography to better handle the trade-off between accuracy and efficiency. Depending on the data processing goals, these approaches can be divided into optimization in the sinogram domain, optimization in the reconstruction domain, and optimization in both domains.

For optimization in the sinogram domain, a common approach is to use the sinogram^67^^,^^68^^,^^69^ directly as input, which better decouples from the reconstruction process. For example, in 2018, Liang et al.^67^ proposed a deep residual convolutional network to recover the sinogram in the projection domain, significantly reducing streak artifacts in subsequent reconstruction. However, due to the large number of projection angles and pixel counts, taking the entire sinogram as input would dramatically increase the data volume and make the training process more difficult. Therefore, in 2018, Lee et al.^68^ introduced a UNet architecture to obtain a lightweight design. The team divided the sinogram into patches of size 50 × 50 pixels and separately fed them to the network to alleviate the burden on random access memory (RAM) during training. The trained UNet achieved good results in metrics like structural similarity index (SSIM) and peak signal-to-noise ratio (PSNR). In 2022, Okamoto et al.^69^ divided the sinogram into band patches and devised an even lighter residual network that successfully augmented the sinogram data and suppressed streak artifacts. Using band patches, more vertically detailed information is attained than using 50 × 50 patches without a significant increase in input volume, which could considerably benefit the sinogram recovery process.

For optimization in the reconstruction domain,^70^^,^^71^^,^^72^ UNet is frequently used. The architectures of these UNets are similar. One classic network, FBPConvNet, was introduced by Jin et al.^70^ in 2017. FBPConvNet takes one of the slices acquired from filtered back-projection (FBP) as input, and through four downsampling and upsampling layers, optimized slices are obtained. The cross-entropy of the optimized slices from the network output and the ground truth is used as the loss function, and then supervised training is performed. In addition to UNet, in 2022, Okamoto et al.^71^ performed streak artifact removal for reconstructed slices using a specialized CNN that incorporates residual blocks and inception blocks^36^ for better feature fusion capability. This model can recover reconstructed images obtained from sparse-view tomography and can also be used for reconstructed image optimization tasks under cone-shaped incidence beams. Furthermore, rather than directly obtaining optimized images, in 2018, Xie et al.^72^ contrived a GoogLeNet-based artifact-learning network. The optimized image is obtained by subtracting the artifacts of the learned image from the original image, similar to Noise2Noise.^73^ Due to the similarities of band artifacts on reconstructed slices, unsupervised methods can be applied to help learn these similarities among slices, remove image artifacts, and improve the quality of subsequent reconstructed images.

For optimization in both domains, sinogram and reconstructed slices can be jointly studied for projection images in sparse-view tomography. In 2023, Gao et al.^74^ proposed an attention-based dual branch (CT branch and sinogram branch) network called ADB-Net. For the sinogram branch, atrous spatial pyramid pooling (ASPP) combined with convolutional layers is applied on sinusoidal images for higher-level feature extraction globally. For the CT branch, the features are fused with the extracted features obtained from the sinogram branch using an attention-based approach through downsampling in a UNet architecture. The strategy strengthens the information critical to sparse-view optimization while weakening the less relevant information. Compared with the optimization technique using a single domain,^70^ ADB-Net showed superior results in terms of root-mean-square error (RMSE), SSIM, and PSNR due to the additional domain. However, due to limitations of the training dataset, ADB-Net still needs to be fine-tuned to obtain optimal performance on new input data. Also, the number of network parameters is enormous because the sinogram utilizes fully connected layers for feature extraction.

In summary, DL is being used for optimization in the sinogram domain, in the reconstruction domain, and in both domains with improved accuracy. The similarity between optimizations of sparse-view tomographic reconstruction and compressed sensing in computer science implies that thoroughly tested models can be directly adopted into synchrotron tomography without major modification.

### Limited angle

During the data acquisition process, certain factors can prevent the acquisition of a full 180° tomography stack at synchrotron beamlines. Such factors include constrained angles arising from sample stage and mechanical facilities or from intrinsic sample characteristics, resulting in limited-angle acquisition. Such acquisition leads to information loss in the wedge-shaped area during tomography reconstruction in the Fourier domain, and the reconstructed slices will also have wedge-shaped artifacts. Effective reconstruction algorithms are therefore needed to recover the reconstructed slices containing these artifacts.

Traditional methodologies, such as the algebraic reconstruction technique (ART)^75^ and simultaneous iterative reconstruction technology (SIRT),^76^ are widely used in limited-angle synchrotron tomography and generally outperform the FBP algorithm. However, with a slow reconstruction process, the drawback of the ART and SIRT algorithms is often their computational efficiency. Therefore, DL approaches are being used to improve reconstruction efficiency through supervised, unsupervised, or self-supervised learning strategies. Exhaustive studies have demonstrated the effectiveness of limited-angle synchrotron tomographic reconstruction using UNet and GAN architectures.^77^^,^^78^^,^^79^^,^^80^^,^^81^

For supervised DL network models, the optimization problem involves domains of reconstructed slices and sinograms. The cross-entropy between the optimized output slices and the ground truth is usually considered as the loss function during the training phase. Reconstruction algorithms, such as FBP^70^^,^^77^ or Simultaneous Algebraic Reconstruction Technique (SART),^78^^,^^79^ can be used with these models, and it is critical to determine which algorithm yields better optimization results. SARTConvNet, a performant network proposed by Wang et al.,^78^ uses the SART algorithm for sinogram reconstruction as well as a classic UNet architecture with BatchNorm added for regularization. This network outperformed other methods^70^^,^^80^ in terms of PSNR and SSIM. DL can also learn to reconstruct optimized slices from a sinogram, where the loss function utilizes the cross-entropy information between the output sinogram and the ground truth during training. GANrec, proposed by Yang et al.^81^ in 2020, is based on GAN architecture and uses a generator network to learn the reconstruction and optimization process. The algorithm was verified on a simulation model extracted from the 3D structure of a shale sample, showing much improved reconstruction accuracy using SSIM and PSNR metrics. In the field of ET, due to limitations regarding the rotation range of the sample stage, supervised DL optimization approaches^82^^,^^83^ on reconstructed slices or sinograms have also been studied, with UNet architecture widely adopted as the generator model of GAN. Wang et al.^82^ effectively recovered data with only a range of −50° to 50° using UNet++ as the generator of GAN, while Xin et al.^83^ achieved good recovery results on missing ET data at 45° by combining reconstructed slices and sinograms using UNet as the GAN generator.

Because paired datasets are not always readily available, and the amount of usable data is often limited, the generalizability of DL networks is becoming a concern. Unsupervised learning can solve the challenge of collecting labeled data and expanding data volume, which could improve generalizability using a massive amount of expanded training data. In 2021, Barutcu et al.^84^ extended the functionalities of GANrec and developed the TomoDIP_TV network by combining the unsupervised deep image prior (DIP).^85^ TomoDIP_TV transforms the reconstruction model at limited angles into a constraint optimization on auxiliary variables during the reconstruction process. The network uses FC layers and 3D convolutions for feature extraction, and the output 3D reconstructed object is obtained following the last convolutional layer. Back-projection is then performed to update the auxiliary parameters until the specified number of iterations is reached. Using DIP to improve the reconstruction, the network implicitly optimizes the weights that recover the missing parts of projections, with assistance from a physical forward model. Compared with GANrec, the network also demonstrates superior robustness to noise. However, due to the self-training strategy used, the model requires another complete training process whenever new data are presented, which is much less efficient than supervised learning methods. Additionally, the implementation of 3D convolutions imposes a significant burden on computational resources.

### Denoising

Denoising on reconstructed images is often considered the most common task in 3D reconstruction optimization. In most *in situ* and dynamic tomography experiments, the exposure time to acquire each projection image is reduced significantly if sparse-view acquisition is not applied, resulting in lower-SNR images that may further decrease reconstruction quality. Therefore, denoising techniques should be applied on reconstructed images to enhance the quality of recovered structures. Similarly, in the medical field, concerns about radiation dose also exist due to the involvement of human subjects. The progress^86^^,^^87^^,^^88^^,^^89^ made so far by researchers in the medical field is expected to benefit synchrotron tomography.

The network models for denoising reconstructed synchrotron tomography images have evolved from supervised^90^^,^^91^^,^^92^^,^^93^^,^^94^ to self-supervised^95^ networks. One of the representative works on supervised denoising is TomoGAN, which was proposed by Liu et al.^90^ in 2020. They adjusted the GAN architecture to focus on finely detailed features in high-frequency content of synchrotron tomography. The generator follows the UNet structure for biomedical image segmentation.^91^ The network was trained using simulated data and verified with both simulated and real-world images, demonstrating significant improvements in SSIM and computation time compared with iterative SIRT algorithms. In addition, in 2023, Yang et al.^92^ applied transformer’s multihead self-attention mechanism to retrieve sinogram features from different viewing angles owing to its capability of extracting interrelation between sequence data. They trained a residual network to remove artifacts using the denoised sinogram and noise-corrupted reconstruction slices. The method achieved better results than iterative reconstruction methods and CNN models,^88^^,^^93^ even at extremely low (5%) dose levels.

Collecting noisy-clean tomographic image pairs can be laborious or even impractical, making self-supervised denoising networks a research hotspot. In 2021, Hendriksen et al.^95^ proposed Noise2Inverse, which uses a CNN to remove noise from reconstructed images without a paired dataset. The method divides each sinogram into subsinograms with equally spaced angles to complete noise learning and denoising. It considers the similarities between the noise distributions of the reconstructed image from one subsinogram and the slices reconstructed from the remaining subsinograms, all using the FBP reconstruction algorithm. The network was compared with SIRT and total variation minimization (TV-MIN) on the TomoBank^96^ experimental dataset, demonstrating superior performance in terms of SSIM and PSNR under various noisy configurations. This study demonstrates the capability of self-supervised learning in denoising synchrotron tomography images.

In the medical area, the challenge of constructing paired noisy-clean images still exists. Hence, self-supervised learning methods have also been well studied. In 2020, Qiu et^86^ al. proposed N2NSR-OCT, which utilizes Noise2Noise to learn the similarities between noise distributions of paired training data, including original OCT images and cropped images. In 2022, Jing et al.^87^ studied a supervised learning framework on low-dose CT (LDCT) denoising using self-attention (SA) modules. The framework does not require any normal-dose CT (NDCT) images as labels and includes SA modules in the architecture of residual encoder-decoder convolutional neural network (RED-CNN),^88^ an encoder-decoder network. In 2023, Selim et al.^89^ used a diffusion model for the unsupervised noise reduction task of LDCT images to achieve highly accurate noise-free image generation with significantly improved runtime.

In summary, several significant studies have been done on denoising synchrotron tomographic reconstruction images. The similarities between synchrotron tomography, medical imaging, and computer vision have allowed the migration of advanced denoising models and algorithms from computer vision into the denoising task on synchrotron tomography reconstruction. Widely adopted models, such as CNN or GAN, have been used, and other models, like Swin-Transformer, may be considered as a more performant baseline for feature extraction. The diffusion model can also be used to surpass the performance of GAN. In the future, these models could bring about impressive denoising results in the field of synchrotron tomography.

## Scientific application-oriented data processing on reconstructed data

**Figure 5:**
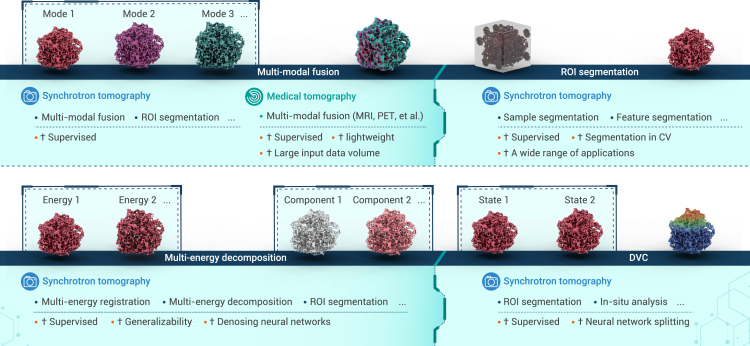
Combination of DL and scientific application-oriented data processing on reconstructed data The versatility of scientific goals leads to a blossoming of DL applications in synchrotron tomography.

Following 3D reconstruction, the scientific application-oriented data processing tasks are closely linked to real-world scientific goals. In the following, we will examine the applications of DL in four areas of scientific application-oriented data processing: ROI segmentation, multimodal analysis, multienergy analysis, and *in situ* tomography (Figure 5).

### ROI segmentation

ROI segmentation is often considered one of the most common scientific application-oriented data processing tasks in the STDPP. The ROI segmentation task is frequently encountered, whether in single-modal or more complex multimodal and *in situ* synchrotron tomography experiments. Due to its similarities to computer vision applications like instance segmentation, DL-based ROI segmentation in synchrotron tomography has been thoroughly studied. Depending on the objects to be segmented, ROI segmentation can be divided into reconstructed intra-voxel segmentation and feature segmentation inside samples.

In some experiments of synchrotron tomography, the true structural distributions are difficult to observe and analyze due to degraded imaging contrast and disturbance from ROIs. Therefore, the segmentation task aims to accurately identify the voxels belonging to highly complex sample structures or partial areas of interest from reconstructed 3D voxels, which would aid in the subsequent analysis process. Numerous efforts have been made, including the use of CNN-based models,^97^ UNet,^98^^,^^99^ and Mask R-CNN.^100^ Here, we focus on the UNet-based approach and the highly accurate Mask R-CNN approach. In 2022, Davydzenka et al.^98^ introduced a typical UNet architecture for highly accurate segmentation of complex Mg-based alloys among reconstructed voxels. In addition to the segmentation advantage of the UNet architecture itself, segmentation results were further improved using a data augmentation method suitable for the tested samples. The augmentation process utilizes randomized sampling and cross-correlation filling methods to expand the current small dataset constrained by a limited number of segmenting labels. For segmentations of composite materials, such a method is efficient and effective because no stringent requirements are raised regarding the continuity of the material itself. Additionally, in 2021, Torbati-Sarraf et al.^99^ performed a comprehensive comparison among UNet, UNet++, PSPNet, and DeepLab v3+ network models. Taking the complex structures of 7075 aluminum alloy (AA7075) as an example, the effectiveness of automatic segmentation on transmission X-ray microscopy (TXM) tomographic data using various models was validated. The basic UNet yields the lowest training time with mediocre performance, while the performance greatly improved for the more complex UNet++^101^ model, which incorporates skip connections. PSPNet^102^ and DeepLab v3+^103^ were pretrained using ImageNet^104^ and achieved decent results, but the performance was still limited. It has been proven that advanced models used for instance segmentation in computer vision can improve the segmentation performance in the field of synchrotron tomography.

For Mask R-CNN,^105^ excellent performance in instance segmentation has been demonstrated in the field of computer vision. For synchrotron tomography, Lin et al.^100^ implemented the Mask R-CNN model for the segmentation of vanadium pentoxide (V_2_O_5_) nanoparticles in 2022. The team encountered the problem of insufficient training data and developed a randomized nanowire generator using Geodict for data generation. They successfully obtained a large amount of simulated training data. Due to the certainties of V_2_O_5_ structures, the model exhibits superior segmentation capabilities on real-world multimodal datasets obtained from X-ray ptychography, STXM, and scanning electron microscopy (SEM) experiments.

In the field of ET, segmentation of the target particle within the imaging area is a significant challenge due to complex cellular structures and the impact of incorporated ice. For DL architectures, common models, like CNN^106^ and encoder-decoder,^107^^,^^108^^,^^109^^,^^110^^,^^111^^,^^112^^,^^113^ are often used. To segment a single particle, some researchers^106^^,^^108^ combined prior knowledge with these DL models to get more easily trained networks. In addition, research on image segmentation using a diffusion model (DM) is being carried out in the medical field. In 2023, Zhang et al.^114^ proposed a generalized hybrid denoising DM (GH-DDM) for cryo-ET images to improve the quality of medical image generation in the absence of high-quality labeled data. Wu et al.^115^^,^^116^ proposed MedSegDiff and MedSegDiff-V2, both with better final testing performance compared with the state of the art (SOTA).

For the task of feature segmentation inside samples, the focus is on segmenting evolving changes in *in situ* synchrotron tomography. Such changes may include features not belonging to intra-voxel information, such as gaps. UNet-based^117^^,^^118^ models are often applied here. In 2021, Kopp et al.^117^ implemented a 5-layer downsampling network architecture for segmenting various types of microscale damage in heterogeneous bulk materials. Additionally, in 2022, Fu et al.^118^ successfully segmented microcracks in an *in situ* lithium-ion battery using a standard 4-layer downsampling UNet. Compared with the 65,000 images required by Kopp et al.,^117^ the team only had 48 images for training and 16 images for validation. Therefore, they expanded the dataset using data augmentation techniques such as rotation, scaling, and cropping. In synchrotron tomography, it is often difficult to attain an immense dataset matching research objectives due to sample complexities. Thus, data augmentation techniques^119^ that expand the size of small datasets are commonly considered effective.

### Multimodal analysis

As the experimental mode for synchrotron tomography is shifting toward multimodal characterization, data fusion between multiple modalities is becoming one of the most challenging steps in the entire STDPP. For example, the fusion of 3D tomography datasets is becoming more difficult due to data throughput explosion at next-generation synchrotron light sources. In synchrotron tomography, conventional and machine learning methods^120^^,^^121^ are still considered the most popular, while DL approaches from other data-intensive scientific fields are also worth investigating. GAN-based architectures have been found to be effective^122^^,^^123^ in performing registration and correlation from different modalities because the end-to-end generative nature of multimodal tomographic data fusion workload is well suited. In 2022, Abirami et al.^122^ used GAN to successfully register single-layer slices obtained from magnetic resonance imaging (MRI) and positron emission tomography (PET) scans, achieving excellent contrast on soft tissues using MRI and better spatial characteristics on bones using PET. The advantages of the two modalities are effectively complemented through modality fusion.

The spatial resolution and FoV for each modality may vary when the tomography datasets are not collected simultaneously, leading to a cross-scale data registration and correlation demand on top of the data fusion. In 2022, Liu and Mukerji.^123^ performed data fusion on carbonate samples obtained by SEM and micro-CT to better understand liquid flows at the porosity level. The structural information covering a wider FoV is obtained from micro-CT, while the finer pore-scale features within a narrower FoV are acquired through SEM. The network used in the study consists of a StyleGAN2^124^ network and a CycleGAN^125^ network. StyleGAN2 generated SEM images close to the ground truth from randomized noise and the style latent vector, while CycleGAN consists of two pairs of generators and discriminators. From the low-resolution micro-CT images, the first pair creates reconstructed images close to the SEM images obtained by StyleGAN; from the reconstructed high-resolution SEM images, the second pair produces reconstructed images close to the low-resolution micro-CT images, which ensures cycle consistency. Eventually, the images from the two scales are fused together using the images from CycleGAN’s SEM generators and micro-CT generators.

It is important to note that collecting paired datasets for multimodal fusion tasks is always considered a challenging task. However, obtaining labels for segmentation is often easier. Therefore, in 2019, Blendowski et al.^126^ transformed the problem of directly fusing multimodal images into the problem of linear interpolation of the shared features across modalities. They used an encoder-decoder network consisting of 3D convolutions to extract information within the shared features of the reconstructed 3D images from various modalities. Later, linear interpolation was applied to complete correlation and registration on a broader scale. Although limited by segmentation quality, the proposed method successfully addressed the problem of paired dataset collection in the complicated multimodal data fusion task. Furthermore, due to the large number of computing resources required by 3D convolutions, which hinders network training and subsequent deployment, Hering et al.^127^ proposed 2.5D convolutions as a substitute for 3D convolutions. The basic idea is to segment the reconstructed voxels into slices oriented toward three directions and compute 3D compensations after proper registration in these directions. This method dramatically reduced the burden on computing resources while guaranteeing registration accuracy.

### Multienergy analysis

Multienergy synchrotron tomography provides extra differentiation capability on material compositions by exploiting the energy dependence of X-ray attenuation for different materials,^128^ following registration and decomposition. In such workloads, the magnification ratios used in tomographic and multimodal tomographic registration tasks are similar because they both involve registration of different magnification ratios, angles, and characterizations. Therefore, the network design should also be similar. Registrations can be performed using UNet or encoder-decoder architectures. For the material decomposition, conventional least-squares dis methods under nonnegative constraints are often applied. Such methods can incur significant noise. Therefore, DL has been adopted to study the decomposition of different materials with high fidelity.

In 2019, Clark et al.^128^ used a simple 2-stage downsampling UNet to separate materials from multienergy CT images. Due to the lack of a real-world dataset, simulated datasets containing iodine, barium, photoelectric effect, and Compton scattering were used to construct the paired training data for supervised training. Using real datasets, the trained network “smoothly” separated iodine and barium with lower noise levels, which would be more difficult using traditional methods. In addition to the basic UNet, deeper networks were also implemented^129^^,^^130^ to further improve decomposition accuracy. For example, in 2020, Gong et al.^129^ applied an encoder-decoder network containing inception blocks.^36^ They retained more preserved structural details while further suppressing noise levels. However, neither network could competently deal with the generalization problem caused by data scarcity. We believe that this problem can be better addressed when the unsupervised learning approaches mentioned under alignment and denoising are considered.

Last, because the challenge induced by noise cannot be effectively addressed solely through conventional methods, denoising the decomposed images using DL could provide an effective solution. Since 2020, Fang et al.^131^ have performed denoising directly on decomposed multienergy data from different materials. They successfully separated distinct materials using the Noise2Noise approach, achieving satisfactory results in terms of detail recovery as well as noise suppression.

### *In situ* tomography

The capability of fast tomography data acquisition in new-generation synchrotron beamlines allows *in situ* characterization of 3D structural and functional evolution processes of samples under various effects caused by force, heat, electricity, magnetic environmental changes, etc. Especially in materials science,^132^^,^^133^^,^^134^ it is critical to quantitatively analyze structural deformation and strain information from reconstructed datasets or segmented features^117^^,^^118^ to identify key functional mechanisms, which turns out to be technically challenging and computationally expensive using conventional algorithms. For example, traditional solutions to 3D deformation characterization usually include the digital volume correlation (DVC)^135^ technique, which calculates internal 3D displacement and strain fields during the deformation process. The technique has been widely adopted in the past 20 years, but due to the numerical iterative process, the computational cost of the entire DVC procedure can still be significant, even with graphics processing unit (GPU) acceleration.^136^ Using the DL solution to address such time-consuming task has just begun. For example, in 2022, Duan and Huang^137^ proposed DVC-Net, which specifically tackles the time-consuming problem caused by numerical iteration. Due to the complexity of DVC, DVC-Net was divided into three subnetworks, with each trained separately. Each subnetwork is a simplified CNN architecture that, respectively, extracts the whole voxel deformation, subvoxel deformation, and “smoothly” acquired displacement fields. The idea of dividing convoluted tasks into subnetworks is noteworthy. Subnetworks present numerous advantages. For instance, training separately using subnetworks could save computing resources, and the problem’s objectives would become more specific with simpler model structures, which benefits the training process. Additionally, collected datasets from other fields highly relate to specific scientific objectives, which would also aid in training. As for results, through the division and combination of three subnetworks, DVC-Net successfully achieved accurate and robust DVC calculations, expediting runtime by two to three orders of magnitude.

## Challenges, opportunities, and outlook

DL is already being widely studied and adopted in the field of synchrotron tomography. It is being used for various tasks, such as distortion correction in image stitching before 3D reconstruction; preprocessing tasks, such as instability mitigation across domains; artifact removal in sparse-view and limited-angle imaging; and 3D reconstruction optimization, such as denoising induced by underexposure; it is also used for complicated and various scientific application-oriented data processing tasks, including ROI segmentation and multimodal, multienergy, and *in situ* tomography. DL has already demonstrated its superiority in data processing accuracy and efficiency and is expected to gradually replace conventional paradigms in the future.

**Figure 6:**
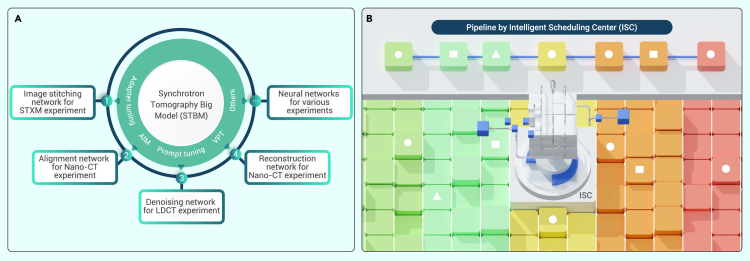
Outlook for DL in synchrotron tomography (A) Different types of networks in downstream tasks are derived through the STBM, using advanced tuning methods. (B) The ISC makes selections from numerous functional DL models to formulate the STDPP.

It should be noted that, due to sample variability and lack of paired data, the feature extraction capability of DL models directly trained from the input end would be limited, resulting in poor accuracy and generalizability in the downstream tasks of the STDPP. Consequently, backbone networks that specifically address the generalizability problem in synchrotron tomography are essential, which is referred to as the synchrotron tomography big model (STBM) (Figure 6A). Using a pretrained STBM in concert with customized downstream networks, better feature extraction results could be achieved for various tasks in the STDPP, making the training process more accurate with less computing resources consumed. For synchrotron beamlines often limited by computing power, STBM is expected to facilitate the development and applications of DL in the STDPP. However, it is still critical to collect an immense amount of data and devise proper network architectures for the STBM. Because synchrotron tomography data are usually categorized into three domains (projection, sinogram, and reconstruction), the corresponding STBMs can be divided into STBM-P, STBM-S, and STBM-R, which all require the acquisition of a large amount of training data in each domain to achieve decent generalizability. Hence, the construction of the training dataset would be more straightforward. In terms of model design, because the ability to learn from a tremendous amount of data is a requisite for the STBM, model prototypes such as ViT, Swin-Transformer, and LDM are considered competent candidates. Moreover, for synchrotron tomography, the main objective of STBM design is feature extraction. Therefore, the training goal could be more flexible with concentration on low-level tasks such as classification, which could eventually ease the training process.

Similar to the big models in computer vision, which contain billions of network parameters, for downstream tasks, directly fine-tuning the entire network using a small dataset could instead downgrade the generalizability and accuracy, let alone the tremendous computational cost induced by a large number of model parameters, which contradicts the initial motivation when designing STBMs. Therefore, the parameters of the STBM should be kept intact when possible. For distinctive downstream tasks, with the parameters of the backbone networks frozen, parameter-efficient fine-tuning (PEFT)^138^ could be applied for training, such as adapter^139^ and adapting image models (AIM).^140^ These plug-and-play models require very few training parameters, making the STBM backbone more suitable for customized downstream tasks in the STDPP without redundant modification. Alternatively, prompt tuning (PT)^141^ and visual PT (VPT)^142^ can be used to fine-tune the STBM by incorporating only a small number of training parameters into the input space after freezing the backbone network parameters. It is also worth noting that soft X-ray tomography based on highly coherent rings and ptychography techniques is also an exciting area that involves more dimensions and richer scientific scenarios, leading to more demand in its data processing pipeline. These tuning techniques enable the derivation of downstream STBM-like models for the soft X-ray tomography^143^ pipeline, which greatly helps reveal multidimensional properties of materials pertaining to structural, chemical, elemental and electronic information.

On the other hand, as the types of models and data scales continue to increase in the STDPP, proper allocation of computing power, as well as the selection of target functional models, is becoming critical for deployment workloads at future large scientific research facilities, such as synchrotron light sources. Limited by the availability, capacity, and capability of computing resources, it is more imperative than ever to create an intelligent scheduling center (ISC) that intelligently allocates computing resources and automatically selects the most suitable models for certain workloads (Figure 6B). Using a pretrained ISC, models residing in the DL library could be autonomously selected and recommended through each stage of the STDPP, which enables the assignment of appropriate pipelines according to distinctive data types and structures for efficient processing and real-time feedback. Also, because the ISC inherently takes into account intermodule correlation and optimal workflows, a graph neural network (GNN) may be used to strengthen such correlation. For optimal workflows, deep reinforcement learning (DRL) can also be applied to formulate the necessary optimal decision strategy. In the training process of DRL, the reward design needs to balance the trade-off between computing power consumption and final tomographic results to reach a “sweet spot.”

Furthermore, a future ISC could be a multimodal model with inputs consisting of images (synchrotron tomography stack) or text (task requirements/metrics) and with outputs consisting of selected pipelines or models. The architecture of contrastive language-image pre-training (CLIP)^144^ could also be used to jointly train a universal ISC big model with data from natural language and machine vision. Later, by making use of the Reinforcement Learning from Human Feedback (RLHF)^145^ technique that is adopted by ChatGPT, the pretrained ISC model should become more adapted to the scientifically more prudent synchrotron tomography area. Like the STBM, finalized optimal designs of the selected pipelines would differ from each other due to differences in task objectives across various beamlines. Therefore, the ISC also needs to be adapted accordingly based on downstream tasks. It would be feasible to adjust ISC models using the “fourth paradigm” of NLP, such as prompt learning, to meet the pipeline design requirements from different beamlines. The goal is to offer a better user experience to synchrotron tomography users and staff and ultimately improve user services in large scientific research facilities.

Besides experimental data, metadata are also worth studying. Metadata can help build single-facility learning schemes for beamline scientists. For example, collected metadata can be used for training using inherent drift data generated at certain synchrotron tomography beamlines. Additionally, due to unpredictable factors, such as mechanical abrasion, the distribution of the objectives to be optimized will gradually change, where a long-term single-facility learning strategy is more desired. By making use of the collected metadata during the elongated experimental process, DL and DRL methods may be applied to learn the trending changes of objective distribution to fit or predict metric variations at synchrotron tomography beamlines. Hence, the STDPP at certain beamlines can be better optimized. Also, enabled by the model’s prediction capability, the system can provide early warnings for beamline instrumentation, such as excessive drift caused by mechanical abrasion.

All in all, whether it is for certain tasks in the STDPP, for the STBM, for an ISC, or for scientific application-oriented data processing on reconstructed tomographic data using single-facility and long-term learning, impacted by today’s data-driven “fourth paradigm,” the full-stack DL-empowered data processing pipeline for synchrotron tomography experiments (STDPP) will be facing unprecedented challenges and opportunities but with an inspiring outlook ahead. The STDPP has all of the potential of being built, deployed, and refined in tomography beamlines, by tomography methodologies, and for tomography experiments at new-generation light sources in the very near future.

## Lead contact website

https://people.ucas.edu.cn/∼zhangyigsxrj.
